# Social Belonging as the Main Concern for Achieving Life Satisfaction When Adapting to Parkinson’s Disease

**DOI:** 10.3390/ijerph18168653

**Published:** 2021-08-16

**Authors:** Lina Rosengren, Anna Forsberg, Christina Brogårdh, Jan Lexell

**Affiliations:** 1 Department of Health Sciences, Lund University, S-221 00 Lund, Sweden; anna.forsberg@med.lu.se (A.F.); christina.brogardh@med.lu.se (C.B.); jan.lexell@med.lu.se (J.L.); 2 Department of Neurology, Rehabilitation Medicine, Memory Disorders and Geriatrics, Skåne University Hospital, S-221 85 Lund, Sweden; 3 Department of Thoracic Surgery, Skåne University Hospital, S-222 85 Lund, Sweden

**Keywords:** adaptation, psychological, Parkinson disease, quality of life, qualitative research, self-management, sense of coherence

## Abstract

Parkinson’s disease (PD) is a complex, progressive neurological condition that impacts daily life and reduces life satisfaction (LS). To achieve and maintain high LS, persons with PD (PwPD) must go through a process of change to adapt to their new life situation. However, our knowledge about this process is very limited. The aim of this study was to investigate the process of change, and the main concern in this process, in PwPD. To study the transitional experience of PwPD, an inductive qualitative approach, using Grounded Theory (GT), was employed. Thirteen participants (9 women, 3 men and 1 non-binary), with a mean age of 54 years (range from 47–62 years), participated in in-depth interviews. Data showed that social belonging is the main concern in the process of change for PwPD. In this process of change, they use strategies to comprehend, accept, adapt, and balance in their strive for social belonging, which in turn can enhance LS. Health care professionals can use this model with an interdisciplinary approach to support PwPD through a successful process of change to achieve social belonging, and thereby achieving and maintaining LS.

## 1. Introduction

One of the most common progressive neurological conditions is Parkinson’s disease (PD). It is caused by degeneration of dopaminergic neurons in the basal ganglia. The degeneration leads to motor symptoms, such as bradykinesia, rigidity, tremor, and postural instability. Additionally, non-motor symptoms, such as fatigue, insomnia, and cognitive impairment, as well as autonomic dysfunction, e.g., orthostatic hypotension [1], are common. PD is a complex condition, and its progression is highly individual. Regardless of the course of progression, an impact on everyday life is inevitable [2]. PD can affect a person’s ability to participate in daily activities [3] and lead to reduced life satisfaction (LS) [4,5,6].

LS is a concept describing the degree to which a person positively evaluates the overall quality of his/her life. It is a global term that refers to both “contentment” and “enjoyment” [7], and is commonly defined as a “global assessment of a person’s quality of life according to his or her own chosen criteria” [8]. LS is dependent on the degree to which one’s expectations are fulfilled [9]. Therefore, to achieve and maintain a high level of LS, a person’s ability to adapt to the changing prerequisites of his/her situation is a key factor [10,11,12]. To increase our understanding of this process, we have explored the meaning of LS and adaptation [13] in persons with Parkinson’s disease (PwPD). Based on qualitative interviews, we described how adaptation to PD involves a transitional process characterized by either acceptance or resistance. Acceptance makes adaption and LS possible, whereas resistance constitutes a behavioral barrier to both adaptation and LS.

From a rehabilitation research perspective, our qualitative study emphasizes the need to understand what underlies adaptation in order to support PwPD to go through this process of change [13]. Thus, we need to understand how this process takes place, in other words—how PwPD go through this process of change when living with PD. By understanding this in greater detail, clinicians can plan more appropriate person-centered rehabilitation interventions for PwPD in order to support their process of change and enable them to achieve and maintain LS over time. However, to the best of our knowledge no study has investigated this process of change in PwPD.

Therefore, the aim of this study was to investigate the process of change, and the main concern in this process, in PwPD.

## 2. Methods

### 2.1. Research Design

This study is part of a project aimed at obtaining a broad and in-depth understanding of LS in PwPD. In our first studies [5,14], we assessed LS from a quantitative perspective, noted the complexity of LS and PD, and recognized the need to gain a deeper understanding of LS in PwPD. Therefore, in our third study [13] we explored LS and adaptation using a qualitative phenomenological hermeneutic approach. The rationale for the present study and the research design chosen is based on the findings from our previous study [13]. The phenomenon of interest in the present study was the transitional experience of PwPD, in other words, what is the main concern during the process of change and how do PwPD deal with it? [13]. Therefore, an inductive approach using Grounded Theory (GT) according to Charmaz [15] was employed.

### 2.2. Participant Selection

The inclusion criteria were adults with a verified diagnosis of PD (ICD10:G20) for at least one year, age below 65 years and being able to participate in the interview. Those with severe cognitive impairment or with major difficulties understanding Swedish were excluded. The recruitment was performed in four steps as described by Morse [16].

1. Convenience sampling. Adults with PD from the regional patient organization in the south of Sweden were recruited in order to identify the scope, major components, and trajectory of the overall process. After five interviews we moved on to the next sampling step.
2. Purposeful sampling. During this step we wished to maximize the variation of meaning. To obtain a nationwide distribution, we approached the national patient organization for PwPD. These five new interviews revealed in a more in-depth manner how the participants themselves were woven into the emerging phenomenon of social belonging. They spoke for themselves and a conceptual scheme, as well as the trajectory identified during the convenience sampling, emerged more clearly. At this stage it became evident that the process starts with a strive to comprehend and that comprehension is the foundation for acceptance that enables adaptation, resulting in the act of balancing.
3. Theoretical sampling. First, we recoded the data in accordance with the identified trajectory and illuminated critical junctures, i.e., the points between the stages. At this stage it was clear that the process went back and forth driven by uncertainty and coherence. We then let the emerging categories and our increasing understanding direct the final sampling. We therefore posed targeted questions to three more participants concerning the meaning of each concept in order to enable grounded definitions as well as clarifying the linkages between the categories. No true negative cases, i.e., participants who did not respond to the emerging categories in the anticipated way, were identified.
4. Validation interviews. Four validation interviews were performed, where we worked deductively and asked the participants if the analysis made sense to them and if there was a match between their experiences and the emerging theoretical model. The model was confirmed, and the participants assisted us in explaining the loop of coherence and uncertainty. Finally, the validation interviews also clarified the link between social belonging and LS.

After including 13 PwPD we were convinced that we understood what we observed, could identify its relevant forms and that it appeared culturally consistent. Therefore, the sampling ceased. A total of three males, nine females, and one non-binary person, with a mean age of 54 years (range 47–62 years) were included in the study. The mean time since diagnosis was 3.4 years. The participants gave their informed written consent and the interviews took place at a location chosen by them: their own home (*n* = 8) or by digital techniques (*n* = 5).

### 2.3. Data Collection

The data collection concluded in 2020. The open-ended interviews were recorded and transcribed verbatim immediately after each interview. Thoughts, emotions, and actions from the time the participants became ill and were diagnosed with PD to the time of the interview were recalled during the interviews. The open-ended questions enabled the participants to more vividly relate memories and elaborate on their experiences. The interviews lasted for a mean of 67 min (range 42–110 min) and resulted in 222 pages of transcribed text. As the interviews were very rich, the data also enabled a separate analysis regarding the meaning of adaptation to PD which is described in our previous qualitative study [13].

### 2.4. Data Analysis

Initially we conducted a literature review as recommended by Hallberg [17] and Glaser et al. [18] in order to establish whether previous studies with a GT approach and similar focus had been carried out, and no such study was found. We performed initial line by line coding to find words or phrases indicating important categories, qualities, or contexts related to the research question [15]. Reflections and questions that emerged during the analysis and coding process were logged for each interview. We then conducted focused coding in order to detect and explain the most frequent and significant codes, which illuminated the main concern experienced by the participants. Theoretical coding indicated relationships between the codes generated from the focused coding and the constant comparative method (CCM) [15] was used simultaneously for data, codes, and categories. Finally, the process of change and the ways the participants dealt with it were identified.

## 3. Results

**Figure 1:**
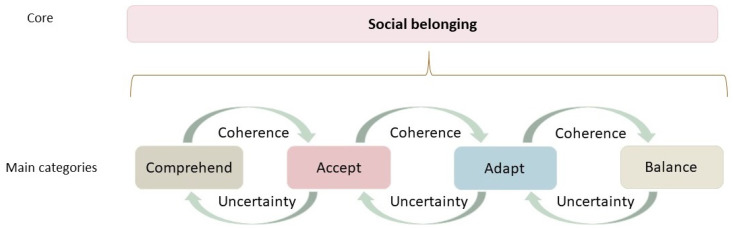
The grounded theory of social belonging among persons diagnosed with Parkinson’s disease.

The core category—social belonging—summarizes a process wherein the generated GT is present within the four main categories: Comprehend, Accept, Adapt, and Balance (Figure 1). Throughout the generated process, a trajectory of achieving social belonging is evident. This starts when the person is diagnosed with PD and continues until balance is reached, and social belonging is achieved. Along this trajectory, the PwPD is moving back and forth between coherence and uncertainty, constituting the theoretical link between the main categories.

Comprehension enables acceptance, which in turn promotes adaptation, as it is easier to adapt when one understands the circumstances, as it is expected that things will work out based on what one has experienced and understood. Sufficient and satisfying balance is achieved when expectations and outcomes are in line with each other. Our results also suggest a relationship between social belonging and experiencing LS. For example, an unwanted side-effect of the medication might be a profound smell of perspiration which would not be a problem if one lacks social contacts or lives alone. But one does not want to smell bad at work, as it causes concerns regarding social belonging. An additional dreaded side effect is gambling addiction that would affect social relations and social belonging. Thus, uncertainty arises when new symptoms occur that demand dose adjustments, leading to the need for comprehension and learning, acceptance, adjustment and balancing in order to maintain social belonging and subsequently LS. When PwPD succeed in achieving sufficient balance to maintain social belonging the level of LS is high.

*“Life is good even if you’re not always functioning.”*

(Validation interview no 1)

The main categories contain several subcategories. Based on the outline of the results presented in Figure 1, subcategories are presented in bold italics in the following text with quotations in italics.

### 3.1. Comprehend

The process starts by trying to comprehend PD, its consequences in everyday life, medication, side effects, and prognosis. To ***seek knowledge*** by reading pamphlets, asking health care professionals, searching the Internet, and attending meetings at the patient organization or social media groups is one important strategy for making sense of the situation. To ***learn*** and ***reflect*** builds experience, which is an important part of coherence. The participants practice learning by doing, learning from others, and learning together with a spouse. Furthermore, a useful strategy is to ***compare*** PD with worse diseases in order to place their own disease in a more favorable light. However, to compare with previous levels of high-performance exercise also creates a painful awareness of the disabling consequences of PD. To ***set*** ***new goals*** and to ***evaluate*** new achievements and performance have become a useful strategy to comprehend and establish coherence as the quality of being logical and consistent. Achieving coherence, i.e., the quality of forming a unified whole, drives the process forward to the next stage of acceptance.

*“You were sad for a couple of days, thinking it is not fair, but I don’t know… I’m a rather practical person and started to find a job and was quite busy with that. I read everything I found and learned that I won’t die from PD. Not like in other situations in life, e.g., a diving accident where it was close… A couple of times I have faced death in my life so that was my first question: How long do you live with Parkinson’s disease?”*

(Interview no 7)

### 3.2. Accept

One part of acceptance is to ***prepare*** yourself for different aspects of the disease, signs, and symptoms, that are inevitably linked with PD. To ***approach*** symptoms and difficulties meant actively accepting lack of sleep, fatigue, difficulty focusing, decreased balance and problems swallowing, and slowly figuring out how to adjust activities in everyday life. Accepting also means to ***share*** the disease and its characteristics with relatives and friends. Their acceptance of one’s new state is a way to ensure social belonging. The informants also decided to ***focus*** on their abilities and possibilities, what they still can do and to ***let go*** of the setbacks. Part of acceptance is leaving the things you cannot control and simply facing the reality you’re in.

*“Before this, you took everything for granted thinking you have your whole life to do what you want, but now I focus more on the small things… I usually say that you should not think of what you can’t do, but think of what you can do. Then you feel better.”*

(Interview no 3)

*“In the beginning I used to think that I was not going to accept this [Parkinson’s disease] and this should not beat me… but then I realized that accepting that you have a disease does not mean that you have given up… no I have not given up, I am going to continue to fight to be able to manage it.”*

(Interview no 5)

### 3.3. Adapt

Living with PD means the need to ***change roles*** in the family. From being the capable person organizing the family, there is a new phase where PwPD need help from their spouses and adult children due to strong sensitivity to stress and the need to be reminded of things when the memory is affected. The disease makes it inevitable to ***adjust*** all aspects of life, i.e., diet, less hours at work and dealing with stressful situations, poorer financial status, and an overall changed life situation including uncertainty, feeling lonely, and sometimes depressed or distressed. To ***exercise*** daily is a vital strategy for mastering the physical disabilities involved in PD and something new compared to the life situation before diagnosis. Exercise leads to increased strength and mobility when the benefits exceed the drawbacks and there is a balance. The medical treatment for PD involves ***scheduling*** the whole day to ensure that the medication has the greatest effect and as few side-effects or setbacks as possible. Scheduling also includes planning your daily activities according to your energy and need for recovery. One part of adaption means to ***interact*** with family and friends, healthcare professionals, and the patient organization to ensure social belonging.

*“I have adapted in numerous ways. I started to join a couple of Facebook groups and I read some blogs. I have really tried hard to comprehend what the disease is all about and the more I understand I realize that it is to a large extent up to me. The things I can do to assist my body to be as healthy as possible regardless of the diagnosis or how it is labelled. It could have been burn out-syndrome or whatever. The thing is what I do to give my body the best possible conditions. It is about my sleep, my diet, my way of dealing with stress, meditation, and so on. It has been a long journey and very interesting to learn about the disease.”*

(Interview no 9)

### 3.4. Balance

To balance your life situation in relation to PD is a way of finding balance in life and achieving the social belonging that you aim for. In order to achieve a balance, it is necessary to ***prioritize***, as PD means being more tired and fatigued than before. Thus, PwPD choose wisely when deciding how to spend time on different tasks and what is important. They ***prevent*** setbacks by accepting that their stress levels are higher and try to rest when possible. Furthermore, they ***choose*** their battles and find joy by looking at the bright side, despite a sometimes heavy symptom burden and many side-effects from the medication.

It is not about ceasing to feel sorry for yourself. Instead, it concerns acknowledging the things that bring energy and hope. A way to decrease uncertainty is to plan ahead and thereby ***control*** the things that are possible to master. Another strategy to achieve balance is to ***create*** new routines and learn different ways of doing things. It is about finding joy in spite of everything or celebrating anyway, opening your eyes to the good things that are working, and create new meaningful activities and situations. Finally, being mindful and staying in the present is a way to ***enjoy*** life as it is, which is possible when you comprehend, accept, adapt, and manage to balance abilities and limitations belonging to you in desired social contexts.

*“I go to the dance class where you are in an environment where healthy people also dance. And we can be there, dancing on our own conditions. Perhaps if you had been to a different place it might not have been such fun. But to be in this context and atmosphere has been very positive because everybody is included and you start by sitting on a chair doing different exercises. Also, the ones in wheelchair or with a walker can join the group and feel the community. And after the dance class, we drink coffee and it is very cozy before everybody goes home to their place. The dance brings so much joy.”*

(Interview no 3)

## 4. Discussion

### 4.1. Reflection on the Findings

In this qualitative study, we have explored the process of change in PwPD and the main concern in their transition is to achieve and maintain LS. We found that the main concern in the process of change is social belonging, which summarizes a process wherein the generated GT is present throughout the four main categories: Comprehend, Accept, Adapt, and Balance. The strive for social belonging was obvious throughout the analysis as the drive for change in their new life situation.

Social belonging, as described by our participants, includes belonging to the family, friends, working life, patient organization, or other kinds of the community. A review [19] on social role functioning in PwPD showed how PD symptoms (e.g., tremor, facial masking, fatigue) interfere with social relationships and some PwPD lose friends when they are diagnosed with PD if they try to hide their symptoms instead of speaking openly about it. Another study has shown that PD may precipitate earlier departure from work life [19]. The compromised social participation is also described by Sjödahl Hammarlund et al. [2], where PwPD wished for increased social belonging.

Comprehend was the first step in our model, which is an essential start of the process of change. Understanding the disease and its consequences is essential for accepting, adapting, and ultimately balancing the new life situation. To be able to comprehend, our participants described that it was important to seek knowledge, learn from each other, compare with others, and to learn together with a spouse. These aspects have been described before in PwPD [20], but not in a model of the process of change. Thus, our results show the importance of comprehension for being able to move forward in this process (i.e., accept) in order to achieve and maintain social belonging and a high LS.

We have previously shown that acceptance enables adaptation, and that resistance constitutes a behavioral barrier to adaptation and LS [13]. In addition, acceptance of the disease is also an important factor for treatment outcomes. A study exploring the lived experience of deep brain stimulation (DBS), showed that the more persons with PD felt alienated by their illness, the more they experienced post-operative self-estrangement [21]. In the present study, we demonstrate how acceptance is necessary to move on and adapt to the new life situation. The role of acceptance for behavioral change is well-known [22]. Accepting the disease has previously been described as a gradual process of going through different emotions from denial, sadness, and anger to slowly accepting and eventually looking toward the future with new hope and positive thoughts at the future. Having a positive mind-set and view of both life and the present situation have been described as important strategies for acceptance [20]. In the present study, the participants described that preparing for different aspects of the disease, approaching the symptoms and difficulties, sharing concerns with friends and family, focusing on possibilities and abilities while letting go of setbacks were important strategies for managing the situation. It has previously been shown that sharing with friends and family might prevent the loss of friends [19], which in turn could strengthen the social belonging for PwPD.

Being able to accept the situation enables a person to adapt. We have previously described the meaning of adaptation for LS [13]. Here, we present the strategies used for this adaptation process. It was obvious from the interviews that adaptation is a demanding process for PwPD. It covers every aspect of life including roles at work and at home, as well as strategies for managing everyday activities. The adaptation comprises physical training to increase and maintain physical function, compensating for physical and mental impairments, scheduling activities in relation to medication and energy in order to optimize functioning, thus eliminating symptoms and obstacles in their surrounding environments. These different approaches are the core of neurorehabilitation, which presuppose an interdisciplinary team [23].

The fourth step in our model describes how PwPD strive to find a balance in their daily life. The balancing comprised balance in activity level, balance in expectations and goals, as well as a mental balance to find peace of mind. The participants described how important it was to plan ahead, create new routines, and to do things that were meaningful and enjoyable in order to achieve balance in life. Thus, balancing is an effort to meet the physical as well as psychological needs. Balancing needs and demands have also been described by Sjödahl Hammarlund et al. [2], who suggested a shift in focus from a biomedical to a need-based approach to facilitate more person-centered care for PwPD. Our findings support their suggestion.

Coherence was the theoretical link making it possible to move forward in the process of change. When persons find the situation logical, consistent, and experience the quality of forming a unified whole, they continue to the next part in their process of change. Our findings are in agreement with the concept Sense of Coherence (SOC) described by Antonovsky [24], and we have previously shown that a strong SOC is related to higher LS in PwPD [5]. Thus, SOC may be one factor explaining why some persons tend to have a successful process of change and adaptation, whereas others struggle with this process.

The other theoretical link—uncertainty—implies that persons have to move backwards in their process of change. This is described as happening when something unpredictable occurs, raising questions about how their social belonging will be affected. When uncertainty occurs, the persons have to, once again, use strategies to comprehend, accept, or adapt to the new situation before being able to recover their balance. During this process, they handle the uncertainty and gain a new perceived coherence, making it possible to move forward again. The need for PwPD to find strategies to handle uncertainty has also been described by Hellqvist et al. [20].

Thus, starting from the time of the diagnosis, PwPD are continuously moving back and forth in their process of change, striving to achieve social belonging. Our data revealed that social belonging made the participants experience high LS, which was also confirmed by the four validation interviews. The similar concept ‘belonging’ and its importance for LS has previously been described in the literature, and also supports our findings [25]. Due to our knowledge of the importance of social belonging for LS, the absence of social belonging could be one explanation for reduced LS in PwPD as the disease progresses [5].

### 4.2. Clinical Implication

The interviews in this qualitative study revealed that the participants used both emotion-focused and problem-focused strategies in order to comprehend, accept, adapt, and balance life with PD. It is important that healthcare professionals support these persons in the various steps of this process of change. During the first step, comprehend, it is important to give PwPD the opportunity to comprehend their new situation before presenting strategies for adaptation. One strategy could be to provide information about the consequences of PD, but also to encourage group activities. At some point, inviting family members could further facilitate the comprehension and serve as a starting point in the process of change before moving on to accepting. To support the next step, accept, healthcare professionals could encourage PwPD to reflect and to share his/her thoughts with friends and family members. PwPD could also be encouraged to join therapy groups or be introduced to patient organizations. Additionally, providing PwPD with Acceptance and Commitment Therapy (ACT) [26] might be useful. ACT is a cognitive-based therapy used to increase psychological flexibility and the perceived satisfaction with life rather than removing unpleasant experiences of life [26]. ACT has been used for persons with other chronic neurological conditions and has shown promising results in improving quality of life in people with multiple sclerosis [27]. Using ACT in PwPD has also been recently suggested by Ngatsu et al. [28]. To support the next steps in the model—how to adapt to the situation and find a balance in life—a goal-oriented individualized interdisciplinary rehabilitation program is important for PwPD. Various goals and interventions could be supported by a team with different rehabilitation professionals.

Furthermore, facilitating social belonging could be a way of enhancing LS in PwPD. This is in line with the study by Takahashi et al. [28], who found that being part of a group was related to higher LS in persons with musculoskeletal impairments. LS could be enhanced by increasing social belonging in the community [29]. Daley et al. have also shown that belonging is protective of LS in persons with disabilities [30]. One clinical implication for increasing LS could therefore be interventions including group-based activities, which might increase social belonging [19,20]. However, each person has his/her own prerequisites for social belonging and LS, which implies that the process must still be highly individual. The management of PwPD should therefore be person-centered and goal-oriented to support the process of change through self-management. Several studies have demonstrated that conventional PD therapy has little positive effect on social role functioning [19], which highlights the need for interdisciplinary multiprofessional rehabilitation as a complement to medical treatment to enable PwPD to go through a process of change. Thus, our GT model can be used as a road map for rehabilitation professionals to find out where the person is in his/her process of change, and thereby plan appropriate person-centred rehabilitation interventions to increase social belonging and, in turn, achieving and maintaining high LS.

### 4.3. Methodological Considerations

We followed the evaluation criteria for rigor in constructivist GT studies presented by Charmaz [15]. Credibility was achieved by including elucidative and descriptive quotations in each main category, while originality was ensured as the study describes the basic process to achieve social belonging among PwPD by means of a novel grounded theoretical model. Resonance is evident in the model, while the main categories illustrate the richness of the participants’ experiences being diagnosed with PD. The study is useful (usefulness) due to the fact that the findings increase awareness of the social process and various strategies adopted to maintain social belonging and subsequently achieve and maintain LS. The core category—social belonging—created a new understanding of the process that takes place to deal with the main concern when living with PD. The main categories—Comprehend, Accept, Adapt, and Balance—represent new concepts that depict the main concerns of PwPD and how they cope with them.

Theoretical saturation was evident after 13 interviews. At the end of the analysis process, the subcategories and main categories confirmed the analysis rather than adding new data. Quotations from the participants illustrate that the theoretical model stems from the coding process and we continuously checked the constructed concepts and the model against the data to confirm and optimize the result. We consider the theoretical model to be relevant not only among the participants in this study, but also among those who recognize themselves in the core category and its process due to the rather generic characteristics of the model. A strength is that we asked four participants to scrutinize and validate the theoretical model to ascertain whether they recognized the experiences described. Their feedback was overwhelmingly positive in terms of confirming the model but also in helping us to refine the details by further clarifying the loop of coherence and uncertainty that constitutes the theoretical link between the main categories.

There were some limitations in this study. It was difficult to recruit men for the interviews, leading to a somewhat uneven gender distribution. Recruiting through patient organizations may be a limitation. However, all the participants generously shared their experiences and provided rich narratives. Since all of them had a mild to moderate PD, were below the age of 65 years, and were Swedish, transferability is limited to other severities of PD, other age groups, and other ethnical and cultural contexts. All interviews were conducted in Swedish, and all quotations were translated to English. The translation may impact the meaning of the narratives, but not necessarily affecting the importance of them. However, all quotations were validated by a native speaking professional English translator to fully capture the original meaning and intention by the participants. Finally, the literature review was informed by Hallberg [17] and Glaser et al. [18], which might be a limitation since there are other references also describing how to approach literature reviews when performing a GT investigation.

## 5. Conclusions

Social belonging is the main concern in the process of change for PwPD. In this process of change, they use strategies to comprehend, accept, adapt, and balance in their strive for social belonging, which in turn can lead to high LS. Healthcare professionals can use this model with an interdisciplinary approach to support PwPD through a successful process of change to achieve social belonging, and thereby achieve and maintain LS.

## Data Availability

All data were archived according to the Swedish Act concerning the Ethical Review of Research Involving Humans to attain confidentiality and are available upon reasonable request.
